# Harnessing a T1 Phage-Derived Spanin for Developing Phage-Based Antimicrobial Development

**DOI:** 10.34133/bdr.0028

**Published:** 2024-03-20

**Authors:** Wakana Yamashita, Shinjiro Ojima, Azumi Tamura, Aa Haeruman Azam, Kohei Kondo, Zhang Yuancheng, Longzhu Cui, Masaki Shintani, Masato Suzuki, Yoshimasa Takahashi, Koichi Watashi, Satoshi Tsuneda, Kotaro Kiga

**Affiliations:** ^1^ Research Center for Drug and Vaccine Development, National Institute of Infectious Diseases, Tokyo 162-8640, Japan.; ^2^ Department of Life Science and Medical Bioscience, Waseda University, 2-2 Wakamatsu-cho, Shinjuku-ku, Tokyo 162-8480, Japan.; ^3^ Division of Infectious Diseases, Advanced Clinical Research Center, The Institute of Medical Science, The University of Tokyo, Tokyo 108-8639, Japan.; ^4^ Antimicrobial Resistance Research Center, National Institute of Infectious Diseases, Tokyo, Japan.; ^5^ Division of Bacteriology, Department of Infection and Immunity, School of Medicine, Jichi Medical University, Shimotsuke-shi, Tochigi 329-0498, Japan.; ^6^ Department of Engineering, Graduate School of Integrated Science and Technology, Shizuoka University, 3-5-1 Johoku, Naka-ku, Hamamatsu, Shizuoka, 432-8561, Japan.; ^7^ Phage Therapy Institute, Comprehensive Research Organization, Waseda University, 2-2 Wakamatsu-cho, Shinjuku-ku, Tokyo 162-8480, Japan.

## Abstract

The global increase in the prevalence of drug-resistant bacteria has necessitated the development of alternative treatments that do not rely on conventional antimicrobial agents. Using bacteriophage-derived lytic enzymes in antibacterial therapy shows promise; however, a thorough comparison and evaluation of their bactericidal efficacy are lacking. This study aimed to compare and investigate the bactericidal activity and spectrum of such lytic enzymes, with the goal of harnessing them for antibacterial therapy. First, we examined the bactericidal activity of spanins, endolysins, and holins derived from 2 *Escherichia coli* model phages, T1 and T7. Among these, T1-spanin exhibited the highest bactericidal activity against *E. coli.* Subsequently, we expressed T1-spanin within bacterial cells and assessed its bactericidal activity. T1-spanin showed potent bactericidal activity against all clinical isolates tested, including bacterial strains of 111 *E. coli*, 2 *Acinetobacter* spp., 3 *Klebsiella* spp., and 3 *Pseudomonas aeruginosa*. In contrast, T1 phage-derived endolysin showed bactericidal activity against *E. coli* and *P. aeruginosa*, yet its efficacy against other bacteria was inferior to that of T1-spanin. Finally, we developed a phage-based technology to introduce the T1-spanin gene into target bacteria. The synthesized non-proliferative phage exhibited strong antibacterial activity against the targeted bacteria. The potent bactericidal activity exhibited by spanins, combined with the novel phage synthetic technology, holds promise for the development of innovative antimicrobial agents.

## Introduction

The emergence of antibiotic-resistant bacteria poses a global threat to public health. In 2019, drug-resistant bacteria were responsible for 1.27 million annual deaths, surpassing the 863,837 deaths attributed to HIV and the 643,381 deaths caused by malaria, making them the leading causes of infectious disease-related mortality [1]. Furthermore, a 2016 review on antimicrobial resistance (AMR) commissioned by the United Kingdom government estimated that, by 2050, 10 million people will die annually as a result of AMR if no action is taken [2]. To address this issue, the World Health Organization and US Centers for Disease Control and Prevention have published a prioritized list of pathogens for AMR drug discovery research. Among the highest-priority pathogens are multidrug-resistant *Acinetobacter* spp., multidrug-resistant *Pseudomonas aeruginosa*, and carbapenem-resistant *Enterobacteriaceae*, including *Escherichia coli* and *Klebsiella* spp. [3,4].

The dwindling number of approved antimicrobial agents since 1990 necessitates exploration of novel therapeutic strategies [5]. Phage therapy, an alternative to traditional antimicrobial therapy, uses bacteriophages (phages) that specifically infect and lyse bacteria, offering potential treatment options for various bacterial infections, including diarrhea and skin infections [6,7]. In recent years, phage-derived antimicrobial proteins have also garnered attention, with numerous bactericidal phage-derived factors reported [7–11]. Notably, endolysins, a class of phage-derived antimicrobial proteins, can lyse bacteria by hydrolyzing the peptidoglycan layer [12]. Research on and development of endolysins against gram-positive bacteria have been particularly advanced [13] due to their unique ability to access the peptidoglycan layer externally. This characteristic enables target bacteria to be lysed by exposure to endolysins. Compared with conventional antibiotics, endolysins minimize the occurrence of AMR and are therefore attracting attention as agents against antimicrobial-resistant bacteria [12–15]. CF-301 is an endolysin-based therapy currently in clinical development that targets *Staphylococcus aureus* bacteremia and infective endocarditis [16]. Clinical studies are underway to investigate its effectiveness at improving cure rates if used as an adjunctive therapy combined with antibiotics. In addition to endolysins, other bacteriolysis enzymes derived from phages include holins and spanins [15]. Holins facilitate puncturing of the inner membrane of bacteria through oligomerization facilitated by a transmembrane domain, whereas spanins mediate fusion between the outer and inner membranes [17,18]. There are 2 types of spanins: the spanin complex, found in the λ phage, and the unimolecular spanin, found in the T1 phage. The spanin complex comprises i-spanin and o-spanin. The N-terminal transmembrane domain of i-spanin is embedded in the inner membrane, whereas o-spanin is anchored to the inner leaflet of the outer membrane by 3 fatty acyl chains. Unimolecular spanins attach to the inner and outer membranes through a C-terminal transmembrane domain and an N-terminal outer membrane lipoprotein. After cleavage of the peptidoglycan layer by endolysin, spanins fuse the inner and outer membranes, leading to the release of cytoplasm and bacterial lysis.

In this study, we conducted a comparative analysis of bacteriolytic enzymes derived from the T1 and T7 *E. coli* phages. We also assessed the bactericidal activity of T1-spanin against a broad range of gram-negative bacteria including carbapenem-resistant *Enterobacteriaceae* and attempted to synthesize phage capsids loaded with T1-spanin to kill the target bacteria. The results of these experiments demonstrate the potent bactericidal activity of phage-derived spanins. We also developed a technique to incorporate spanins into phage capsids to kill target bacteria.

## Materials and Methods

### Bacterial strains and culture conditions

The bacterial strains used in this study are listed in Tables S1 and S2. The bacterial strains were grown at 37 °C in Luria-Bertani (LB) medium (BD Difco, Franklin Lakes, NJ, USA). Unless otherwise indicated, appropriate antibiotics were added to the growth medium at a final concentration of 100 μg/ml for carbenicillin (Crb), 30 μg/ml for kanamycin (Km), and 34 μg/ml for chloramphenicol (Cm).

### Measurement of lysogenic enzyme expression

*E. coli* MC1061 transformed with arabinose-inducible red fluorescent protein (RFP) expression plasmids was cultured overnight in 2 ml of LB+Cm medium with shaking at 200 rpm. Subsequently, 700 μl of the overnight culture was diluted in 70 ml of LB+Cm medium and incubated for 1 hour with shaking at 200 rpm. The culture was divided into six 10-ml portions, and different concentrations of glucose and arabinose were added, resulting in the following final concentrations: glucose 0.2 wt%, arabinose 0, 0.01, 0.05, 0.1, and 0.2 wt%. The RNA in each culture was extracted using the miRNeasy Mini Kit (Qiagen, Hilden, Germany), subjected to DNase treatment using DNA-free DNA Removal Kit (Thermo Fisher Scientific, Waltham, MA, USA), incubated at 65 °C for 5 min and finally gDNA was removed and reverse transcribed to create cDNA using ReverTra Ace qPCR RT Master Mix with gDNA Remover (Toyobo Co. Ltd., Osaka, Japan). Samples without RT were created as controls. Each sample was added to a 96-well plate and a real-time polymerase chain reaction was performed using THUNDERBIRD Next SYBR qPCR Mix (Toyobo) and specific primers. StepOnePlus Real-Time PCR Systems (Thermo Fisher Scientific) was used for measurements and calculation of Ct values. A resistance marker gene (*cat*) was used as an internal control, and the *rfp* gene expression value was divided by the *cat* expression value for normalization.

### Spot test

*E. coli* MC1061 harboring arabinose-inducible gene expression vector (pKLC23), 23-RFP, 23-T1 endolysin, 23-T1 holin, 23-T1 spanin, 23-T7 endolysin, 23-T7 holin, and 23-T7 spanin were streaked on LB+Cm plates and incubated overnight at 37 °C. Four colonies from each plate were selected and inoculated into 2 ml of LB+Cm+glucose (Glu) (0.2 wt%) medium, followed by incubation at 37 °C with shaking at 200 rpm for 6 h. Next, 100 μl of each culture was transferred to the first row of wells of a 96-well plate and used as the original solution. Serial dilutions were performed from 10^−1^ to 10^−7^ using LB+Cm medium. Next, 4 μl of each dilution was spotted onto LB+Cm+Glu (0.2 wt%), LB+Cm, and LB+Cm+arabinose (Ara) (0.2 wt%) plates. The plates were then incubated at 37 °C, and photographs were taken after 12 h. An incubation time of 12 h was selected because the colonies were visible after 12 h. Satellite colonies began to emerge if the incubation period was extended.

### Growth assay

*E. coli* MC1061 harboring pKLC23, pKLC23-T1 endolysin, pKLC23-T1 holin, pKLC23-T1 spanin, pKLC23-T7 endolysin, pKLC23-T7 holin, and pKLC23-T7 spanin were streaked on LB+Cm plates and incubated overnight at 37 °C. Four colonies from each plate were collected and incubated overnight with 2 ml of LB+Cm+Glu (0.2 wt%) medium at 37 °C with shaking at 200 rpm. The cultures were then diluted 100-fold with LB+Cm+Glu (0.2 wt%), LB+Cm, and LB+Cm+Ara (0.05 wt%, 0.1 wt%, and 0.2 wt%), and 200 μl of each dilution was added to separate wells in a 96-well plate. Additionally, 200 μl of LB+Cm+Glu (0.2 wt%), LB+Cm, and LB+Cm+Ara (0.05 wt%, 0.1 wt%, and 0.2 wt%) were added as blank controls. The plates were incubated at 37 °C with shaking at 600 rpm, and optical density (OD) at 600 nm (OD600) was measured every 10 min for 24 h. To determine the kinetics of bacterial growth in *E. coli* MC1061 harboring pKLC23 containing each lytic enzyme from bacteriophages, OD600 was measured in LB+Cm+Ara (0.2 wt%). *E. coli* MC1061 strains harboring each lytic enzyme were streaked on LB+Cm+Glu (0.2 wt%) plates and incubated overnight at 37 °C. A colony from each plate was collected and incubated overnight with 2 ml of LB+Cm+Glu (0.2 wt%) medium at 37 °C with shaking at 200 rpm. The cultures were then diluted with LB+Cm to OD600 = 0.5 and centrifuged at 7,000 × *g* for 2 min. The pellets of each strain were resuspended with LB+Cm+Ara (0.2 wt%), LB+Cm+Glu (0.2 wt%), or LB+Cm. Next, 200 μl of each resuspended strain was added to separate wells in a 96-well plate. The plate was incubated at 37 °C with shaking at 600 rpm, and OD600 was measured every 10 min for 12 h.

### Minimum inhibitory concentration test

*E. coli* MC1061 harboring pKLC23, pKLC23-T1 endolysin, pKLC23-T1 holin, pKLC23-T1 spanin, pKLC23-T7 endolysin, pKLC23-T7 holin, and pKLC23-T7 spanin were streaked on LB+Cm+Glu (0.2 wt%) plates and incubated overnight at 37 °C. Colonies from each plate were collected into 2 ml of phosphate-buffered saline (Wako, Osaka, Japan), and the density of each strain was adjusted to the 0.5 McFarland standard preparation. Next, 10 μl of each sample was inoculated into 200 μl of LB+Cm+Ara medium in a round-bottom 96-well plate. Each strain was cultured under arabinose conditions at final concentrations of 0.00325 to 3.2 wt% to induce the expression of lytic enzymes. The minimum inhibitory concentration (MIC) of arabinose in each strain was measured.

### Confirmation of ribosome-binding site strength in plasmid vectors

Ribosome-binding site (RBS) variants were constructed using PCR to create RFP expression vectors (pKLC83-RFP RBS1 to 4 constructs) with different RBS sequences (RBS1 to 4). *E. coli* MC1061 cells were transformed with these plasmids and incubated at 37 °C with shaking at 200 rpm for 4 h. Fluorescence and absorbance (OD600) were measured using a plate reader. The fluorescence intensity of each RBS variant was divided by bacterial cell density.

### Specific sterilization of S17-1 bacteria

*E. coli* S17-1 is a conjugative donor strain harboring the *tra* gene on its chromosome, thereby facilitating the transfer of genetic materials containing the oriT (origin of transfer) through the mechanism of conjugation. In this study, we used S17-1 harboring *λpir* to reduce the number of copies of mobile plasmid within S17-1. The S17-1 strain containing pKLC276 and pKLC83 (control) without lytic enzyme and 2 *E. coli* clinical isolates were plated on LB and LB+Crb plates. Colonies were incubated in 2 ml of LB+Crb/LB medium overnight at 37 °C with shaking at 200 rpm. Next, 100 μl of each culture was transferred to a 96-well plate and serially diluted in LB in concentrations ranging from 10^−1^ to 10^−7^. Subsequently, 4 μl of each dilution was spot-tested on plates containing LB and M9 minimal media (1.5 wt% agar, 0.2 wt% arabinose, 0.1 mM CaCl_2_, and 2 mM MgSO_4_) at an arabinose concentration of 0.2 wt%. The plates were then incubated overnight at 37 °C, and photographs were taken to assess the growth and survival of the bacterial strains.

### High-throughput assay to investigate bactericidal activity of T1-spanin and T1-endolysin

A donor strain, S17-1 containing pKLC276 (a Tet repressor and arabinose-inducible T1-endolysin) and pKLC263 (an arabinose-inducible gene expression vector), pKLC263-T1-spanin, or pKLC263-T1-endolysin was plated on LB+Crb+Cm+Glu plates and incubated overnight at 37 °C. The colonies were selected and incubated in LB+Crb+Cm+Glu medium overnight at 37 °C with shaking at 200 rpm. Clinical isolates were plated and incubated overnight at 37 °C. The colonies from clinical isolates were suspended in LB in 96-well plates and incubated for 6 h at 37 °C with shaking at 600 rpm. Overnight cultures of S17-1 cells were centrifuged at 6,000 × *g* for 3 s at room temperature and resuspended in LB. A new 96-well plate was prepared, and 100 μl of each S17-1 strain and clinical isolate was added to each well. The plates were cultured at 37 °C for 20 h. After diluting the culture in LB medium, spots were placed on M9 minimal broth and LB+Cm plates. After overnight incubation at 37 °C, the plates were photographed.

Similar investigations were conducted with *P. aeruginosa* and *Acinetobacter* spp., using gentamicin (Gm) instead of Cm as the antibiotic. The bactericidal activity of lytic enzymes was assessed by counting colonies on M9 minimal broth+Cm plates. Strains that did not grow when spread on LB+Cm plates, could not be sterilized on LB+Cm medium, or exhibited extremely poor growth on M9 medium were excluded from the analysis.

### Synthesis of T1-spanin-loaded phage capsids

*E. coli* 594 carrying pKLC83 and either pKLC296 or pKLC296-T1-spanin and incorporating the φ80 genome (without the packaging site) (19) were cultured in 2 ml of LB+Cm+Crb medium for 6 h. The culture was then diluted 100-fold in LB+Cm+Crb to an OD of 0.1. Mitomycin C (1 μg/ml) and arabinose (0.2 wt%) were then added. The cultures were grown overnight. The overnight culture was passed through a 0.45-μm filter to obtain a phage solution. Then, 5 μl of phage solution (φ80(pKLC296) or φ80(pKLC296-T1-spanin)) and 500 μl of an overnight culture of MC1061 or MC1061(pKLC83) were mixed and incubated for 10 min at room temperature. Next, 100 μl of this mixture was added to 3 ml of soft agar and spread evenly on LB+Cm agar plates. After overnight incubation, the colonies were counted.

### Plasmid construction method

DNA was amplified using the primers listed in Table S3 and Q5 High-Fidelity DNA Polymerase (New England Biolabs, Ipswich, MA, USA) and assembled using NEBuilder HiFi DNA Assembly Master Mix (New England Biolabs). The pKLC23-T1-endolysin/T1-holin/T1-spanin/T7-endolysin/T7-holin/T7-spanin plasmid, used for comparison of lysogenic enzymes, was made from pKLC23-RFP as a template and the lysogenic enzymes of the T1/T7 phage were inserted. The pKLC83-RFP (RBS1) was created by assembly of pKLC83 and the RFP region of pKLC23-RFP. pKLC83-RFP (RBS2 to 4), 3 plasmids with different RBS, were created by PCR using pKLC83-RFP (RBS1) as a template and primer with the mutation in the RBS position. For conjugation, pKLC276, 263, and 263-T1-spanin were used. pKLC276 was created by assembling pKLC83 and the endolysin (arabinose-inducible) region of pKLC23-endolysin. pKLC263 was created by removing unnecessary terminator and promoter regions by Nde I from pKLC261. pKLC261 was created using artificially synthesized pKLC260 as a template and inserting the mobile region of artificially synthesized pKLC259. pKLC273 was created by replacing RFP in pKLC83-RFP (RBS3) with T1-spanin derived from pKLC23-T1-spanin, and pKLC279 was created by inserting the Tet-inducible T1-spanin of pKLC273 into pKLC263. Tet repressor was removed from pKLC279 to make pKLC263-T1-spanin. The pKLC296 plasmid used for constructing the phage with T1-spanin was created by assembling pKLC263 and the oriT-containing region of pKLC31. The pKLC296-T1-spanin was created by assembling the non-Tet repressor region of pKLC279 and the oriT-containing region of pKLC31. The plasmids used are listed in Table S4.

## Results

### Comparison of bactericidal activity of lytic enzymes

**Fig. 1.**
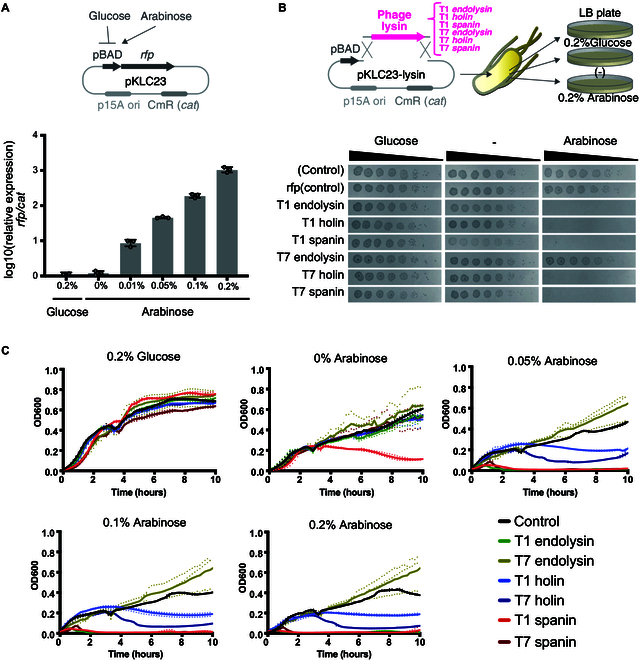
Comparison of bactericidal activity of lysins from bacteriophages. (A) Expression efficiency of genes under the control of the arabinose promoter. *E. coli* MC1061 harboring arabinose-inducible *rfp* expression plasmid (pKLC23) was cultured in LB+Cm medium containing glucose or arabinose. mRNA was extracted and expression of the *rfp* gene was measured using real-time PCR. The data are the means ± standard deviations based on 3 wells per group. (B) Spot test to quantitatively assess the bactericidal activity of phage-derived lytic enzymes. *E. coli* MC1061 harboring pKLC23 plasmid expressing lytic enzymes (endolysin, holin, or spanin) derived from T1 or T7 bacteriophages under the control of an arabinose-inducible promoter grown in LB+Cm+glucose (0.2 wt%) medium were serially diluted from 10^−1^ to 10^−7^ using LB+Cm medium. Four microliters of each dilution was spotted onto LB+Cm+glucose (0.2 wt%), LB+Cm, and LB+Cm+arabinose (0.2 wt%) plates. Spot tests were performed using 4 clones in each *E. coli* MC1061 strain. The photograph shown is the representative data. All assays were replicated 3 times. (C) Growth curves of the *E. coli* MC1061 harboring plasmid expressing lytic enzymes under the control of an arabinose-inducible promoter. Bacteria were cultured in LB medium with a glucose concentration of 0.2 wt% or arabinose with a concentration of 0 wt% to 0.2 wt%. The optical density (OD600) was measured every 10 min for 10 h. The experiment was conducted using 3 independent bacterial cultures (represented by dashed lines), and the average values were plotted as solid lines. Abbreviations: Cm, chloramphenicol; LB, Luria-Bertani medium.

To compare the bactericidal activity of lytic enzymes derived from phages (holin, endolysin, or spanin), we constructed expression plasmids containing these genes under the control of arabinose-inducible promoters [19]. In this study, we focused on 2 model phages of *E. coli*, namely, the T1 and T7 phages, which are characterized by a particularly simple genetic arrangement of the lytic enzyme-gene cluster. Given the potential future applications of lytic enzymes in engineered phages or as antibacterial therapeutic agents, we decided to investigate the antibacterial activity of each gene individually (Fig. 1A). As a first step, we confirmed the activity of the arabinose-inducible promoter in a plasmid evaluating the activity of the lytic enzyme. The *rfp* gene was integrated downstream of the arabinose-inducible promoter on the pKLC23 plasmid and was subsequently transformed into *E. coli* MC1061. Real-time PCR was used to evaluate the expression levels of the *rfp* gene and the AMR-marker gene (chloramphenicol acetyltransferase, cat), which were controlled by the Gm 3'-acetyltransferase promoter in *E. coli* MG1655, within the pKLC23 construct. In the system in which lytic enzymes were cloned under an arabinose-inducible promoter instead of *rfp*, none of the enzymes had an inhibitory effect on bacterial growth in the presence of glucose. However, in the presence of arabinose, the expression of T1-holin, T1-endolysin, T1-spanin, T7-holin, and T7-spanin provided evidence of inhibition of bacterial growth (Fig. 1B). To quantitatively assess the bactericidal activity of the phage-derived lytic enzymes, we added 0.2 wt% glucose or varying concentrations (0 wt%, 0.05 wt%, 0.1 wt%, or 0.2 wt%) of arabinose to the bacterial culture media and measured the bacterial growth rate (Fig. 1C). Notably, strong bactericidal activity of T1-endolysin, T1-spanin, and T7-spanin was observed in the presence of 0.05 wt% arabinose. Furthermore, T1-spanin displayed bactericidal activity even without the addition of arabinose. These findings suggest that T1-spanin is a bactericidal enzyme capable of killing bacteria even at low expression levels.

Next, the kinetics of bacterial growth were determined under the expression of T1-holin, T1-endolysin, T1-spanin, T7-holin, T7-endolysin, and T7-spanin. When the lytic enzymes were induced with 0.2% arabinose, the bactericidal kinetics of T1-spanin, T1-endolysin, and T7-spanin were nearly identical, completing lysis within 2 h (Fig. S1). In contrast, T7-holin required approximately 7 h for lysis. T1-holin and T7-endolysin did not exhibit marked lytic activity. Next, we investigated the MIC of arabinose in *E. coli* harboring arabinose-inducible T1-holin, T1-endolysin, T1-spanin, T7-holin, T7-endolysin, and T7-spanin plasmids at final concentrations ranging from 0.00325 to 3.2 wt%. The T1-spanin plasmid exhibited the highest bactericidal activity (MIC 0.025 wt%), followed by the T7-spanin plasmid (MIC 0.05 wt%) and the T1-endolysin plasmid (MIC 0.1 wt%). The T1-holin plasmid and T7-endolysin plasmid displayed low bactericidal activity (MIC >3.2 wt%) (Table 1).

**Table 1.** Minimum inhibitory concentration (MIC) of arabinose for inducing lysins

	Name of lysin					
	T1 endolysin	T1 holin	T1 spanin	T7 endolysin	T7 holin	T7 spanin
MIC (arabinose%)	0.1	>3.2	0.025	>3.2	0.20	0.05

### Development of a high-throughput method for evaluating bactericidal activity of target genes against clinical isolates

**Fig. 2.**
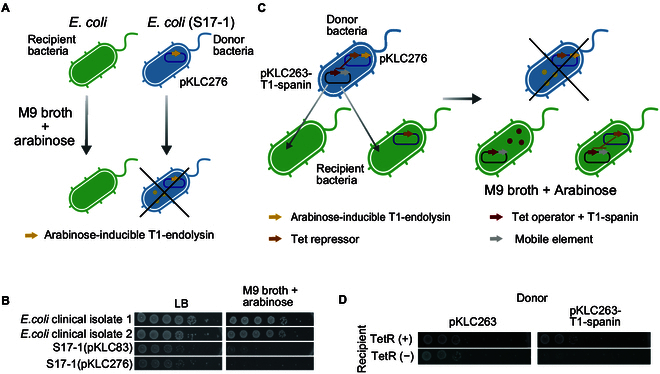
Development of a method to investigate bactericidal activity of target genes using conjugation. (A) Schematic diagram of a selective killing method for the donor strain *Escherichia coli* S17-1, using M9 minimal broth and T1-endolysin. (B) Selective killing of the donor strain S17-1. Clinical isolates of *E. coli*, and the donor strains, *E. coli* S17-1(pKLC83) or *E. coli* S17-1(pKLC276 [arabinose-inducible endolysin]), were spotted onto agar plates containing LB medium or M9 minimal broth supplemented with 0.2 wt% arabinose. (C) Illustration of a conjugation system used to assess the bactericidal activity of T1-spanin. The donor *E. coli* strain S17-1 (blue) carries a Tet repressor expression plasmid (required for suppressing the expression of T1-spanin within the donor *E. coli*) and a T1-spanin expression plasmid. Through conjugation, the mobile T1-spanin expression plasmid is transferred to recipient bacteria (green). The mixed bacteria are cultured on the agar plate containing M9 minimal medium supplemented with arabinose, leading to the killing of the donor bacteria. T1-spanin is expressed in recipient cells that do not express the Tet repressor. (D) Recipient bacteria, expressing the Tet repressor encoded by pKLC83, received a tetracycline-inducible T1-spanin expression plasmid (pKLC263-T1-spanin) through conjugation. The bacterial culture was spotted onto agar plates containing M9 minimal medium supplemented with arabinose. The photograph shows representative data. All assays were performed in triplicate. Abbreviation: LB, Luria-Bertani medium.

To assess the bactericidal activity of the target proteins, specifically T1-spanin, which exhibited the highest bactericidal activity, we established a high-throughput evaluation method that uses a tetracycline-inducible promoter and bacterial conjugation system (Fig. 2A). In the tetracycline-induced system, the Tet repressor binds to the operator sequence and represses the expression of downstream genes [20]. However, upon the addition of tetracycline, the operator is released from the Tet repressor, inducing the expression of downstream genes. We transformed a mobile plasmid containing the Tet operator sequence and another plasmid encoding the Tet repressor into S17-1 *E. coli*, which facilitates plasmid DNA transfer through conjugation [21]. During the construction of this system, we observed that S17-1 strain, which expressed T1-spanin under the Tet-inducible promoter, did not grow well, even when its expression was repressed by the Tet repressor. To address this issue, we weakened the RBS sequence for T1-spanin under the Tet-inducible promoter. We replaced RBS1 (iGEM BioBricks: BBa_B0034) (5′-AAAGAGGAGAAA-3′) with RBS3 (iGEM BioBricks: BBa_B0031) (5′-TCACACAGGAAACC-3′), resulting in a reduction in gene activity to approximately 1/60 (Fig. S2) [22]. Subsequently, to enable a high-throughput assessment of conjugation efficiency, we devised a system to selectively eliminate the S17-1 strain after conjugation. To achieve this, conjugated samples were cultured in M9 minimal medium, which does not support the robust growth of nutrient-requiring S17-1. As we observed a slight proliferation of S17-1 even in M9 minimal medium, we further expressed T1-endolysin from an arabinose-inducible promoter to completely inhibit its growth (Fig. 2B). Next, to demonstrate that the growth inhibition observed in recipient bacteria carrying plasmids expressing the lytic enzyme was attributable to the lytic enzymes and not to conjugation, we investigated the conjugation of the T1-spanin plasmid using a strain in which the expression of T1-spanin was inhibited by the Tet repressor (Fig. 2C). No significant difference in the efficiency of conjugation was observed in recipient bacteria in which T1-spanin was inhibited by the Tet repressor (Fig. 2D). In contrast, when *E. coli* lacking the Tet repressor was used as a recipient of T1-spanin plasmid, the number of colonies was significantly reduced compared with that of *E. coli* with the Tet repressor (Fig. 2D). These results indicate that the presence of lytic enzymes on mobile plasmids do not have a significant effect on conjugation efficiency, and the constructed system can be used as a tool to investigate bactericidal activity of lytic enzymes.

### Bactericidal activity of the T1-spanin against clinical isolates

A total of 142 clinical isolates of *E. coli*, 7 clinical isolates of *Klebsiella* spp., 17 clinical isolates of *P. aeruginosa*, and 16 clinical isolates of *Acinetobacter* spp. were prepared to assess the bactericidal activity of T1-spanin (Table 2). In order to investigate lytic enzyme activity, we adopted a system that involved comparing bacterial lethality by lytic enzymes after the selection of drug-resistant markers following conjugation (Fig. 2D). Hence, the clinical isolates examined needed to be susceptible to antibiotics. Antibiotic susceptibility tests were conducted, and bacteria demonstrating resistance to either Cm or Gm were selected (Fig. S3). Clinical isolates showing resistance to both were excluded. Furthermore, in this assay system, the growth of clinical isolates was evaluated using M9 minimal medium, which suppresses the growth of S17-1. As there was one strain of *E. coli* that did not grow in M9 minimal medium, it was excluded. Additionally, if clinical isolates produced bacteriocins or similar substances, there was a risk of killing S17-1. To address this, S17-1 and clinical isolates were co-cultured, and strains in which S17-1 survived were selected. Among these, 14 strains of *E. coli*, 5 strains of *P. aeruginosa*, and 1 strain of *Acinetobacter* spp. inhibited the survival of S17-1. Moreover, some clinical isolates exhibited low conjugation efficiency from S17-1, likely due to restriction–modification systems or plasmid incompatibility. These strains were excluded, resulting in a final selection of 111 strains of *E. coli*, 3 strains of *K. pneumoniae*, 3 strains of *P. aeruginosa*, and 2 strains of *Acinetobacter* spp. for the evaluation of lytic enzyme activity.

**Table 2.** Characteristics of clinical isolates and selection of strains for conjugation assay

Recipient bacteria	Number of strains					
	Number of clinical isolates examined	Drug resistance marker did not function	Poor growth in M9 minimal medium	Recipient strain killed donor strain	Low conjugation efficiency	Number of strains used in conjugation assay
*Escherichia coli*	142	9	1	14	7	111
*Klebsiella* spp.	7	3	0	0	1	3
*Pseudomonas aeruginosa*	17	4	0	5	5	3
*Acinetobacter* spp.	16	5	0	1	8	2

**Fig. 3.**
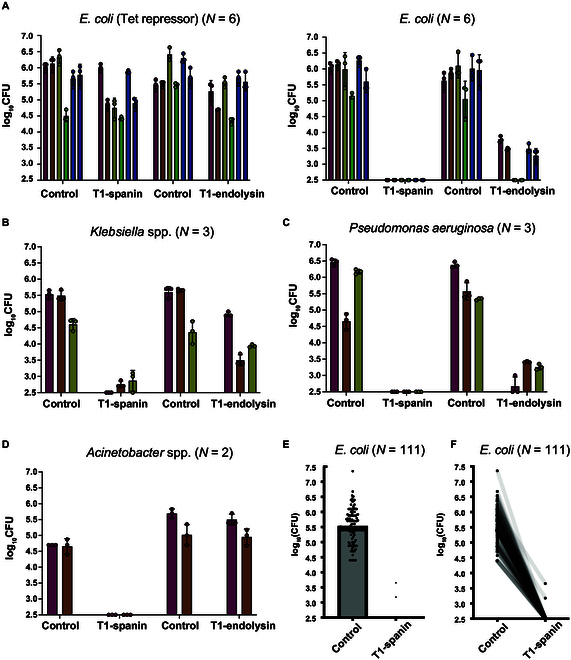
Bactericidal activity of T1-spanin and T1-endolysin against gram-negative bacteria. (A to F) Investigation of bacteriophage lytic activity against clinically isolated gram-negative bacteria. T1-spanin and T1-endolysin were transformed into clinical isolates (*Escherichia coli*, *Klebsiella* spp., *Pseudomonas aeruginosa*, and *Acinetobacter* spp.) as recipient strains by conjugation. For the clinical isolates of *E. coli*, a strain expressing the Tet repressor (which suppresses T1-spanin/T1-endolysin expression) was also prepared. Dilution series of the conjugated samples were prepared and spotted in 4-μl aliquots onto agar plates containing arabinose-supplemented M9 minimal medium. The resultant colonies were counted and plotted. All assays were performed in triplicate. The bars show the means of the 3 spot test results, and the error bars show the standard deviations.

First, we investigated the functionality of a system to evaluate the bactericidal potential of T1-spanin and T1-endolysin against clinical isolates. To this end, clinical isolates of *E. coli* expressing the Tet repressor were constructed, and a plasmid carrying tetracycline-inducible lytic enzymes was transferred to recipient bacteria through conjugation. Consequently, both the control plasmid lacking lytic enzymes and the plasmid encoding lytic enzymes formed nearly an equal number of colonies to the clinical isolates with Tet repressor (Fig. 3A). This indicates that both plasmids can be transferred to the clinical isolates of *E. coli* through conjugation, and the presence or absence of lytic enzymes on the plasmid does not affect the efficiency of conjugation. Next, we investigated the bactericidal activity of T1-spanin and T1-endolysin against 6 *E. coli*, 3 *Klebsiella* spp., 3 *P. aeruginosa*, and 2 *Acinetobacter* spp. strains (Fig. 3A to D and Fig. S4). In the assessment of clinical isolates, all strains that expressed T1-spanin were killed. T1-endolysin also showed growth inhibitory effects against *E. coli*; however, its effectiveness was weaker than that of T1-spanin (Fig. 3A). The bactericidal activity of T1-endolysin against *Klebsiella* spp. strains was notably limited and *P. aeruginosa* was weaker than that of T1-spanin (Fig. 3B and C). *Acinetobacter* spp. were resistant to T1-endolysin (Fig. 3D). Additionally, when the number of clinical isolates of *E. coli* used in the bactericidal test with T1-spanin was increased to 111, T1-spanin showed bactericidal activity against all the strains tested (Fig. 3E and F). These findings indicate that T1-spanin possesses strong bactericidal activity against various types of gram-negative bacteria including carbapenem-resistant *Enterobacteriaceae*.

### Construction of T1-spanin-based bactericidal agents

**Fig. 4.**
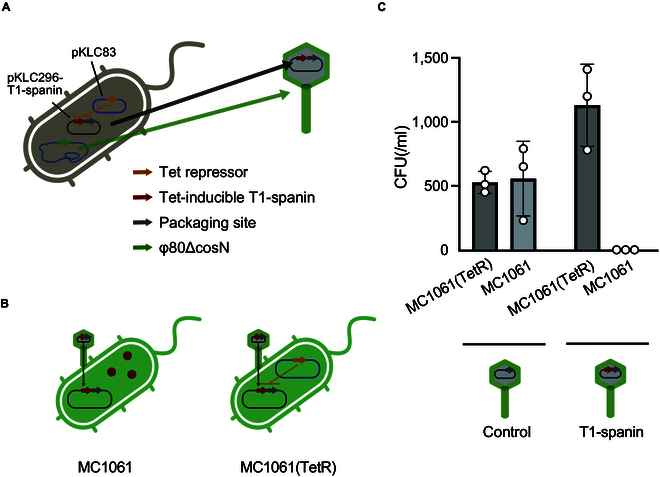
Construction of T1-spanin-loaded capsids and its bactericidal efficacy. (A) Schematic diagram of the construction of a φ80 phage capsid containing T1-spanin gene. T1-spanin exhibits toxicity toward synthetic bacterium 594; therefore, its expression was suppressed using a Tet repressor-expressing plasmid (pKLC83). Due to the presence of a packaging sequence, the T1-spanin plasmid was encapsulated within a phage capsid. (B) The synthesized phage capsid (A) was used to infect *E. coli* MC1061. Within bacterial cells lacking Tet repressor expression, T1-spanin was expressed. (C) *E. coli* MC1061 were infected with phage capsids containing either the T1-spanin-encoding plasmid or a plasmid lacking T1-spanin (control). The infected bacteria were plated on LB agar plates, and the number of colony-forming units (CFUs) was counted. Abbreviation: LB, Luria-Bertani medium. Assays were performed in triplicate. The bars show the means of the 3 spot test results, and the error bars show the standard deviations.

Because spanins exert their bactericidal action by disrupting cell membranes inside bacteria, their expression within bacterial cells is necessary for them to function as antimicrobials. In this study, we used the gene delivery mechanism of bacteriophages to construct a non-replicative phage-based T1 spanin antimicrobial agent for bacteria (Fig. 4A). *E. coli* 594 with bacteriophage φ80 was transformed with a Tet repressor expression plasmid (pKLC83) and a tetracycline-inducible T1-spanin expression plasmid (pKLC296-T1-spanin). The pKLC296-T1-spanin plasmid carries the packaging machinery of the phage-inducible chromosomal island, enabling the packaging of the plasmid into the capsid of φ80 [23]. As the packaging site of φ80 is deleted from the *E. coli* 594 genome, the φ80 genome is not incorporated into the phage capsid during phage production, resulting in a non-replicative phage. T1-spanin expression was repressed by the Tet repressor in the phage-producing bacteria, thereby suppressing its bactericidal activity (Fig. 4A). However, when the plasmid was transferred to strains lacking the Tet repressor, T1-spanin was expressed (Fig. 4B). Phage φ80 (pKLC296), which does not carry T1-spanin, did not exhibit bactericidal activity (Fig. 4C). In contrast, φ80 (pKLC296-T1-spanin) completely killed the MC1061 *E. coli* strain. Furthermore, φ80 (pKLC296-T1-spanin) failed to kill MC1061 (TetR), a bacterium harboring the Tet repressor, indicating that the cell death observed in MC1061 was caused by T1-spanin.

## Discussion

This study demonstrated that phage-derived spanins exhibit potent antibacterial activity against various gram-negative bacteria including carbapenem-resistant *Enterobacteriaceae* and showed that incorporating spanins into phage capsids can serve as powerful antibacterial agents.

Given the diversity of clinical isolates, it is difficult to introduce DNA to a wide range of clinical isolates in a high-throughput manner by phage-mediated transduction or electroporation [24]. In this study, we found that conjugation could be applied to various drug-resistant clinical isolates, making it possible to comprehensively examine the bactericidal activity of the target genes. However, there are some limitations to the system that we developed. First, some clinical isolates kill S17-1 directly. In this study, 14 of 142 strains of *E. coli*, 5 of 17 strains of *P. aeruginosa*, and 1 of 16 strains of *Acinetobacter* spp. killed S17-1. It is likely that the *E. coli* clinical isolates were colicin-producing strains [25]. The *P. aeruginosa* and *Acinetobacter* spp. strains that killed S17-1 might have produced bacteriocins, or might have killed S17-1 through the type VI secretion system [26]. Second, some strains were significantly less efficient at receiving plasmids by conjugation. Possible reasons include failure to maintain mobile plasmids in the recipient strains, inhibition of conjugation due to restriction–modification systems of the recipient strains, or the inability of the needle of the conjugation system to reach the recipient strains due to specific membrane structures.

Important gram-negative drug-resistant bacteria in modern clinical settings, such as *E. coli*, *Klebsiella* spp., *P. aeruginosa*, and *Acinetobacter* spp. [1], were highly susceptible to the bactericidal effects of T1-spanin. Although the host range of T1-spanin has not been fully characterized, T1-spanin functions by fusing the outer and inner membranes of bacteria, and therefore, cannot kill gram-positive bacteria that lack an outer membrane. T1-spanin is a type of bacteriolytic enzyme that is effective from within bacteria, but in the T1 and T7 phages studied here, it has a broader antibacterial spectrum and is more effective than endolysin, a potential therapeutic bacteriolytic enzyme. Therefore, any type of delivery system that transports T1-spanin into bacteria may be a candidate for producing novel antimicrobial agents. It is noteworthy that the bacteria killed by the expression of T1-spanin are resistant to conventional antibiotics. However, comparing the antimicrobial effects of topical antibiotics with those of T1-spanin is challenging because T1-spanin needs to be expressed within the bacterial cell to exhibit lytic activity. To address this issue, we are currently exploring methods to enable extracellular action of T1-spanin by incorporating a membrane-permeable domain into T1-spanin. T1-spanin killed all the gram-negative bacteria tested in this study, but the control T1-endolysin used in this study did not kill the *Klebsiella* spp. or *Acinetobacter* spp. isolates tested. Although both *Klebsiella* spp. and *E. coli* belong to the Gammaproteobacteria class, they have differences in their cell membrane compositions. *Klebsiella* spp. has fewer lipids and thicker cell walls than *E. coli*, and this could have influenced the sensitivity of *Klebsiella* spp. to endolysin [27]. *Acinetobacter* spp., which contains a higher amount of lipids in its cell membrane, might be more sensitive to endolysins.

In this study, we synthesized an antimicrobial agent by incorporating the T1-spanin gene into the capsid of a template phage φ80. This antimicrobial agent demonstrated excellent bactericidal activity against the target bacteria. Furthermore, the synthesized phage is non-replicative, providing the advantage of not spreading into the environment. The phage capsid constructed in this study is based on φ80, and therefore, it shares the same host range as φ80. By modifying the tail region of the capsid, it is possible to alter the host range [28]*.* This capsid synthesis approach can be extended to other phages, allowing for the synthesis of phages targeting other bacteria in addition to *E. coli*. Considering the broad inhibitory effect of T1-spanin on various gram-negative clinical isolates, we posit that the synthetic capsid could serve as an antimicrobial agent capable of selectively eradicating the desired bacterial species. This study serves as an example of antimicrobial agent development using phage capsids harboring potent bactericidal genes. When used as a therapeutic agent, conventional phages can proliferate within bacteria and potentially disrupt the ecological balance. However, after infection and lysing, the phage capsid generated in this work is not self-replicating and does not generate daughter phages. Furthermore, neither horizontal gene transfer nor the phage capsid gene is present in this artificial phage capsid. As a result, there is very little chance that the phage capsid will spread or transfer genes into the environment, and it will have little impact on the environment. In the future, we plan to elucidate the antimicrobial therapeutic effects and the safety of the synthesized phage through animal experiments. Ultimately, we aim to make the method widely applicable against gram-negative bacteria that are resistant to conventional antibiotics.

In this study, we developed a phage synthesis method using antimicrobial factors and evaluated its bactericidal efficacy. Initially, we assessed the bactericidal activity of spanins, endolysins, and holins derived from T1 and T7 phages. Notably, T1-spanin exhibited the highest bactericidal activity against *E. coli*. Bacterial cells expressing T1-spanin demonstrated potent bactericidal activity against various clinical isolates, including *E. coli*, *Acinetobacter* spp., *Klebsiella* spp., and *P. aeruginosa*. Subsequently, a phage-based technology was developed to introduce the T1-spanin gene into target bacteria, resulting in an antibacterial effect. These findings highlight the potential of phage synthetic technology and spanins for the development of innovative antimicrobial agents. This research contributes to addressing drug-resistant bacteria and exploring alternative therapeutic approaches.

## Data Availability

The data that support the findings in this study are available from the corresponding authors on reasonable request. The bacteria list, plasmid list, and primer list are available in the Supplementary Materials.

## Supplementary Materials

Supplementary 1 Figs. S1 to S4 Tables S1 to S4 References [29,30]
