# Supplementary material for: Harnessing a T1 Phage-Derived Spanin for Developing Phage-Based Antimicrobial Development
Source: Biodes Res. 2024 Mar 20;6:0028. doi: 10.34133/bdr.0028 (PMC10954549; doi:10.34133/bdr.0028)
Supplement: Supplementary 1 — Figs. S1 to S4 Tables S1 to S4 References [29,30] [file bdr.0028.f1.zip › Fig.S1.pdf]

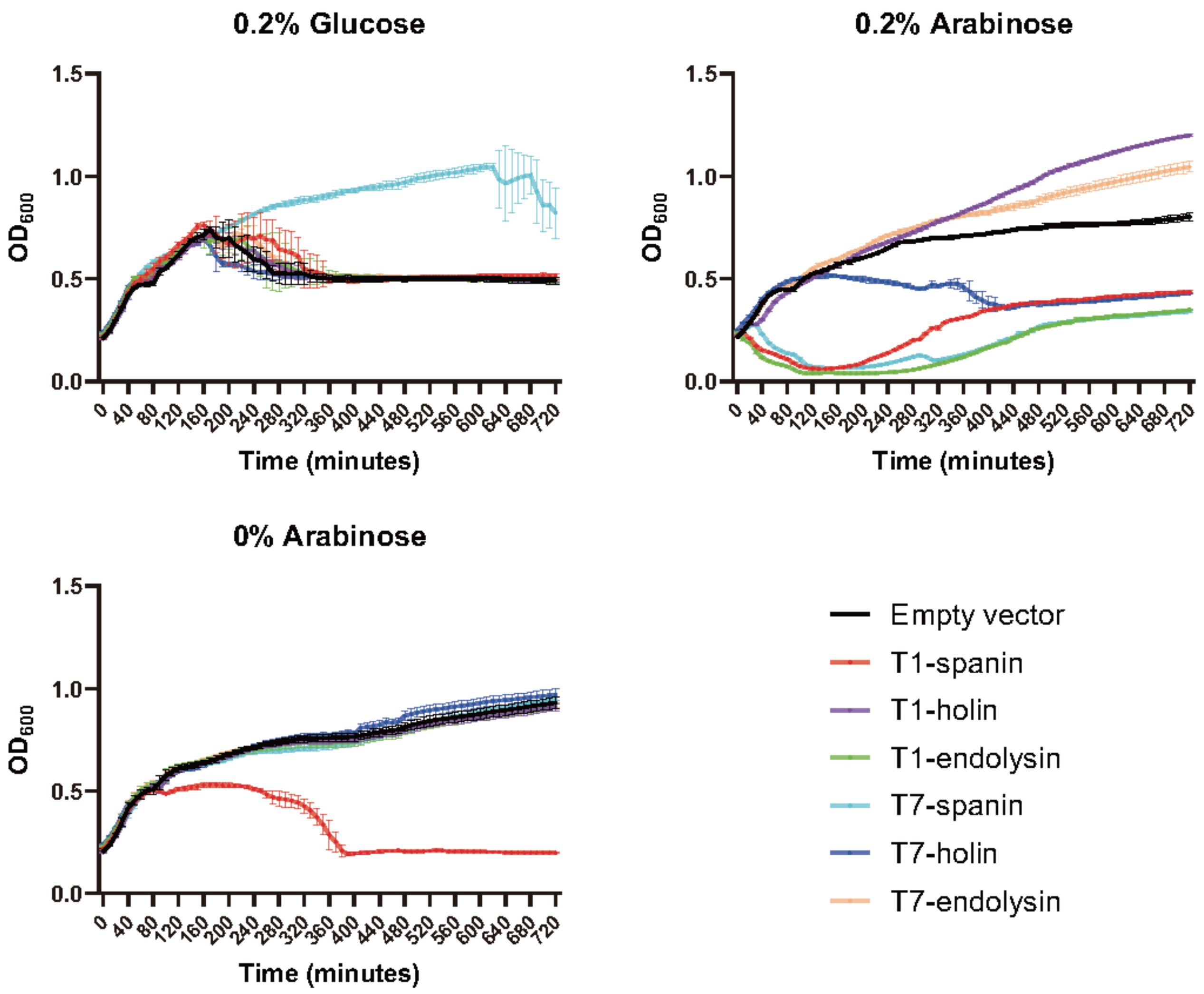

Fig. S1. Comparison of bacterial growth kinetics under expression of lysins from bacteriophages.

The growth kinetics of the *Escherichia coli* MC1061 harboring pKLC23 plasmid expressing lytic enzymes (endolysin, holin, or spanin) derived from T1 or T7 bacteriophages under the control of an arabinose-inducible promoter. Bacteria were cultured in Luria-Bertani (LB) medium with a glucose concentration of 0.2 wt% or arabinose with a concentration of 0 wt% to 0.2 wt%. The optical density (OD600) was measured every 10 minutes for 12 hours. The experiment was conducted using four independent bacterial cultures, and the mean values were plotted as solid lines.
