# Supplementary material for: Harnessing a T1 Phage-Derived Spanin for Developing Phage-Based Antimicrobial Development
Source: Biodes Res. 2024 Mar 20;6:0028. doi: 10.34133/bdr.0028 (PMC10954549; doi:10.34133/bdr.0028)
Supplement: Supplementary 1 — Figs. S1 to S4 Tables S1 to S4 References [29,30] [file bdr.0028.f1.zip › Fig.S2.pdf]

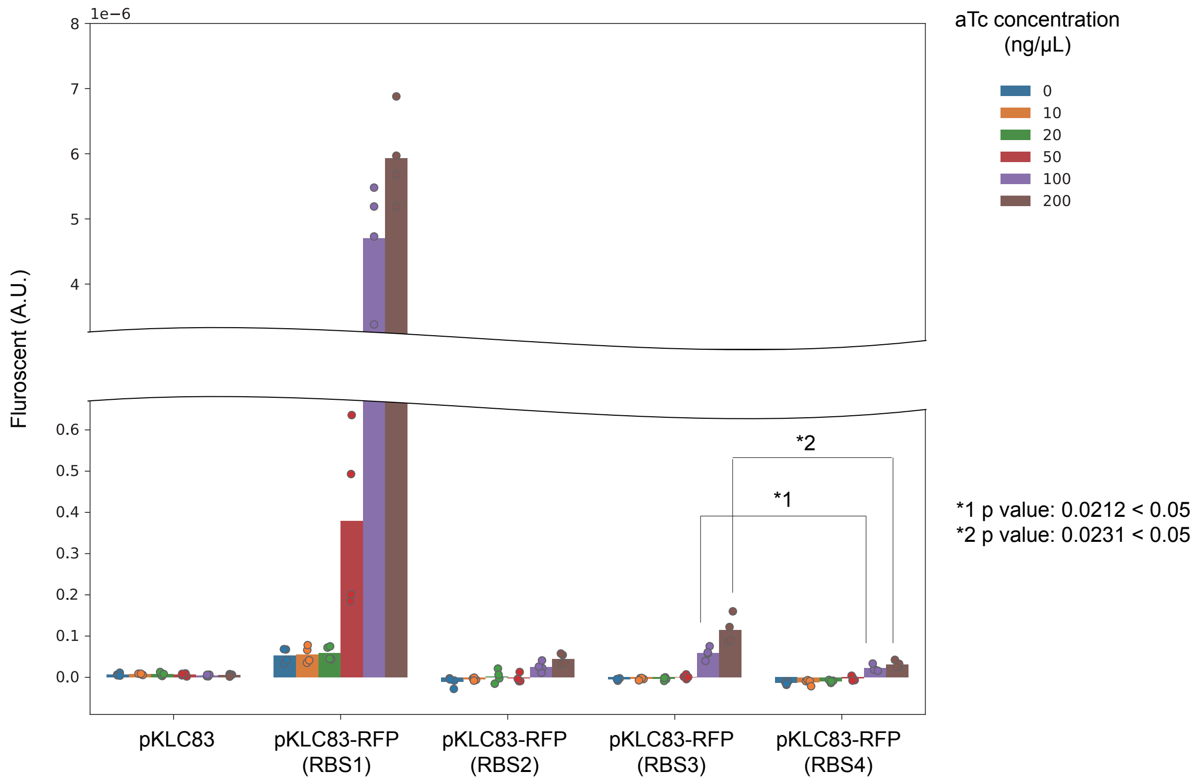

Fig. S2. Comparison of ribosome-binding site (RBS) strength in plasmid vectors under treatment of anhydrotetracycline (aTc).

To confirm the RBS-binding strength of the plasmid vectors, we constructed RFP expression vectors (pKLC83-RFP RBS1–4 constructs) with various RBS sequences (RBS1–4) induced by aTc. These plasmids were transformed into E. coli MC1061 cells and incubated at 37 ° C with shaking at 200 rpm for 4 hours under treatment of aTc (final concentrations: 0, 10, 20, 50, 100 and 200 ng/μL). The fluorescence and absorbance (OD600) were measured using a plate reader. The fluorescence intensity of each RBS variant was divided by bacterial cell density. The data are presented as the means ± standard deviations based on three wells per group. Significant differences are shown as * P <0.05 (t-test). Abbreviations: aTC, anhydrotetracycline; OD600, optical density at 600 nm; RBS, ribosome-binding site; RFP, red fluorescent protein.
