# Supplementary material for: Harnessing a T1 Phage-Derived Spanin for Developing Phage-Based Antimicrobial Development
Source: Biodes Res. 2024 Mar 20;6:0028. doi: 10.34133/bdr.0028 (PMC10954549; doi:10.34133/bdr.0028)
Supplement: Supplementary 1 — Figs. S1 to S4 Tables S1 to S4 References [29,30] [file bdr.0028.f1.zip › Fig.S3.pdf]

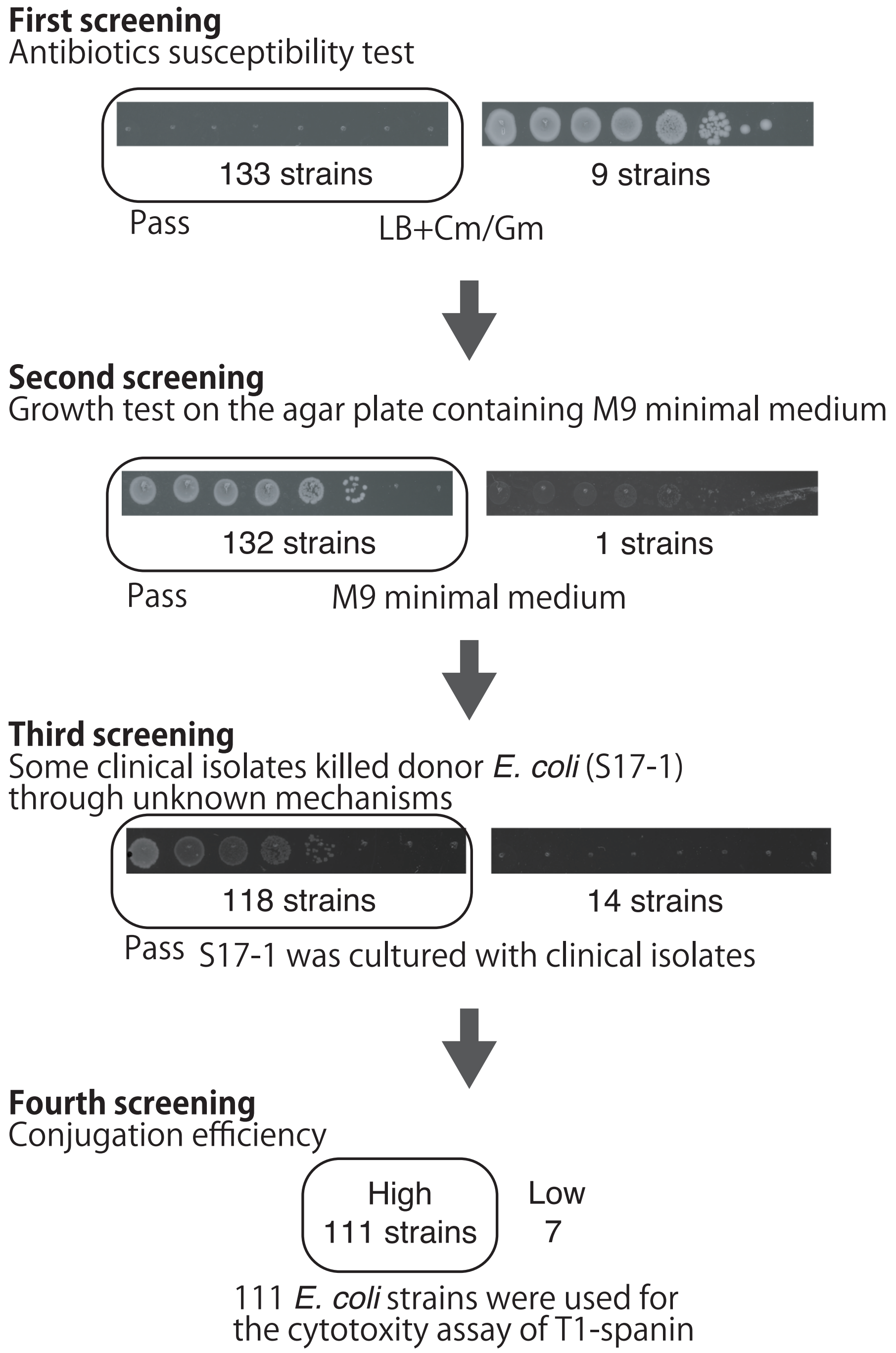

Fig. S3. The process of selecting clinical isolates for evaluating the bactericidal activity of T1-spanin is shown using the example of *Escherichia coli*.
In the first screening, an antibiotic susceptibility test was performed to select bacteria that were resistant to either chloramphenicol (Cm) or gentamicin (Gm), and clinical isolates that were resistant to both were excluded. In the second screening, the growth of the clinical isolates was evaluated in M9 minimal medium, which was used to inhibit the growth of S17-1. Clinical isolates that showed inhibited growth in M9 minimal medium were excluded. In the third screening, clinical isolates were co-cultured with S17-1 to evaluate the viability of each strain. Fourteen strains showed bactericidal activity against S17-1. Finally, a fourth screening was performed to select bacteria that underwent conjugation and had at least 100-fold more colonies than the control bacteria using a spot assay. Of the 142 clinical isolates of *E. coli*, 111 were used for the bactericidal evaluation assay of T1-spanin.
