# Supplementary material for: Harnessing a T1 Phage-Derived Spanin for Developing Phage-Based Antimicrobial Development
Source: Biodes Res. 2024 Mar 20;6:0028. doi: 10.34133/bdr.0028 (PMC10954549; doi:10.34133/bdr.0028)
Supplement: Supplementary 1 — Figs. S1 to S4 Tables S1 to S4 References [29,30] [file bdr.0028.f1.zip › Fig.S4.pdf]

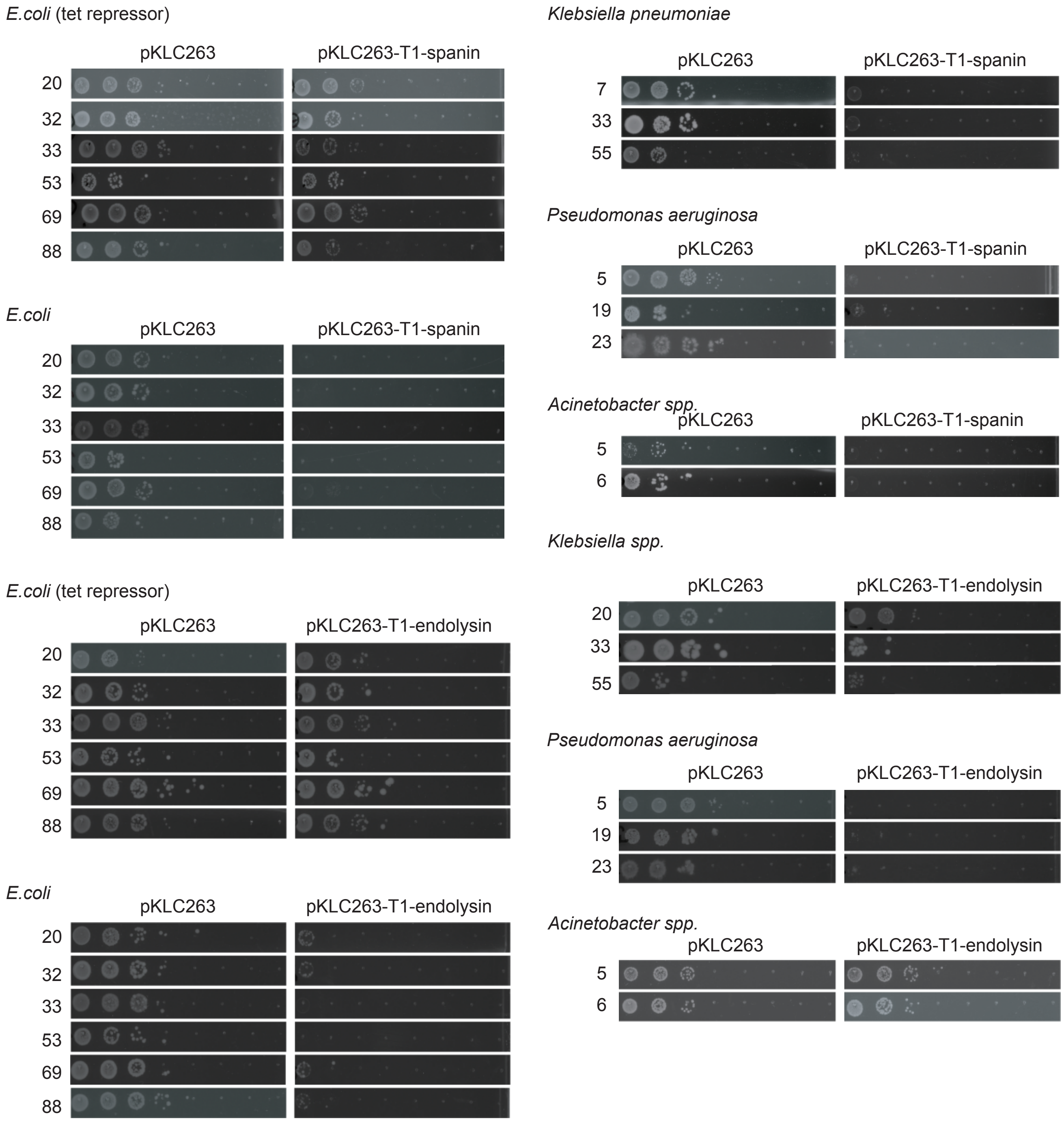

Fig. S4. Culture plates showing the results of T1-spanin and T1-endolysin killing studies. Clinical isolates, including *E. coli*, *Klebsiella* spp., *P. aeruginosa*, and *Acinetobacter* spp., were co-cultured with *E. coli* S17-1 carrying plasmids with T1-spanin or T1-endolysin (pKLC263 series) and serially diluted. The host bacteria were then spotted onto M9 minimal broth+Cm medium to examine the killing activity of T1-spanin and T1-endolysin. Abbreviation: Cm, chloramphenicol.
