# Supplementary material for: Harnessing a T1 Phage-Derived Spanin for Developing Phage-Based Antimicrobial Development
Source: Biodes Res. 2024 Mar 20;6:0028. doi: 10.34133/bdr.0028 (PMC10954549; doi:10.34133/bdr.0028)
Supplement: Supplementary 1 — Figs. S1 to S4 Tables S1 to S4 References [29,30] [file bdr.0028.f1.zip › FigS1-4.pdf]

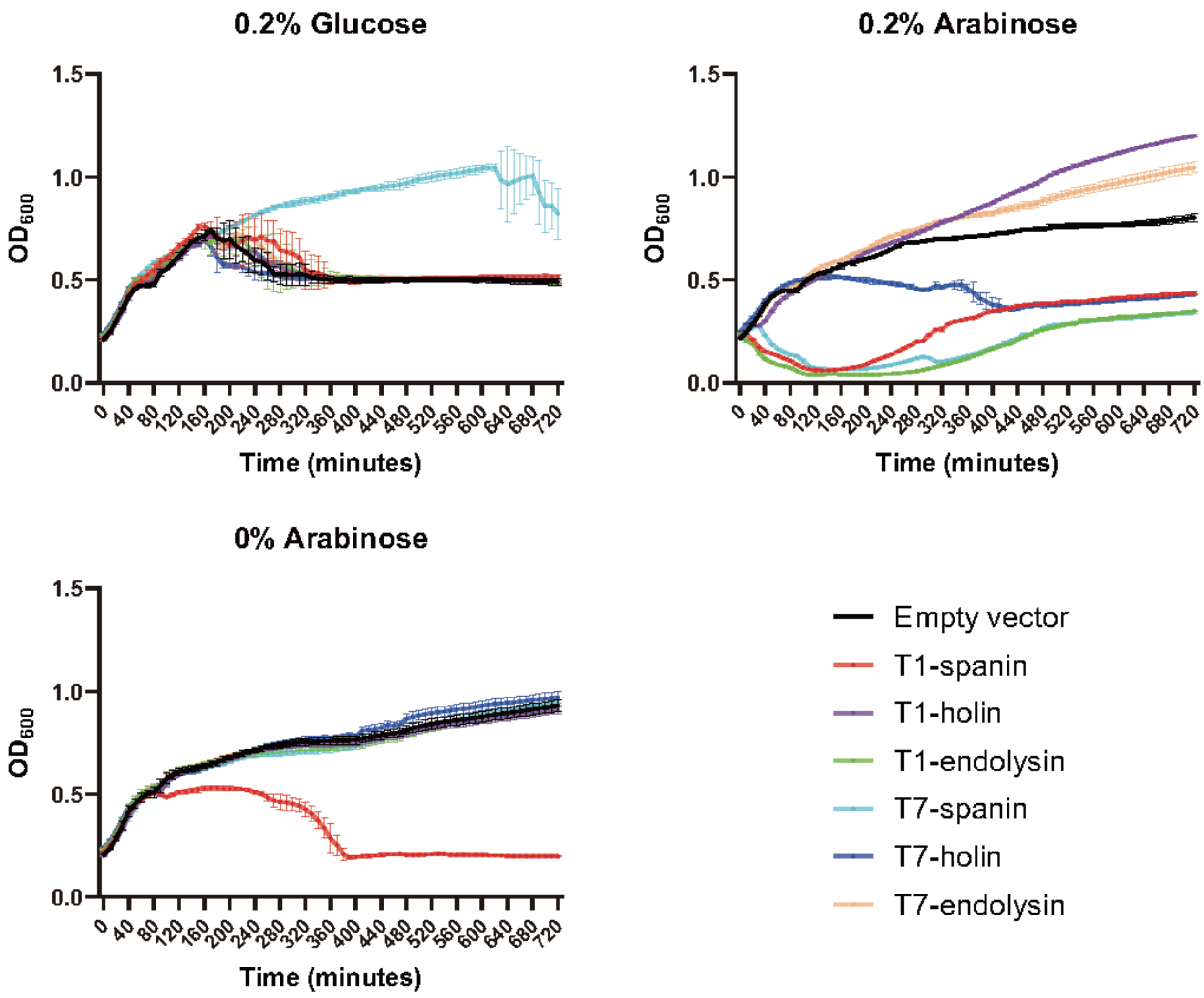

Fig. S1. Comparison of bacterial growth kinetics under expression of lysins from bacteriophages.

The growth kinetics of the *Escherichia coli* MC1061 harboring pKLC23 plasmid expressing lytic enzymes (endolysin, holin, or spanin) derived from T1 or T7 bacteriophages under the control of an arabinose-inducible promoter. Bacteria were cultured in Luria-Bertani (LB) medium with a glucose concentration of 0.2 wt% or arabinose with a concentration of 0 wt% to 0.2 wt%. The optical density (OD600) was measured every 10 minutes for 12 hours. The experiment was conducted using four independent bacterial cultures, and the mean values were plotted as solid lines.

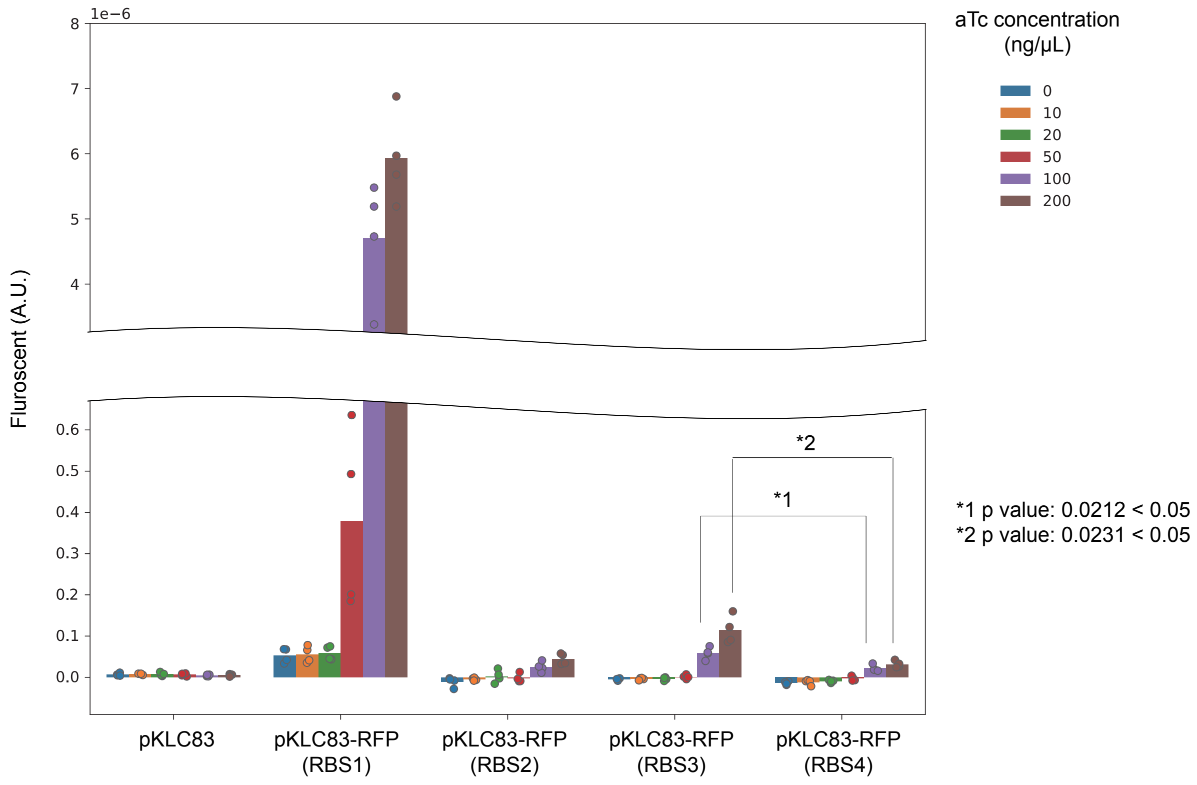

Fig. S2. Comparison of ribosome-binding site (RBS) strength in plasmid vectors under treatment of anhydrotetracycline (aTc).

To confirm the RBS-binding strength of the plasmid vectors, we constructed RFP expression vectors (pKLC83-RFP RBS1–4 constructs) with various RBS sequences (RBS1–4) induced by aTc. These plasmids were transformed into E. coli MC1061 cells and incubated at 37 ° C with shaking at 200 rpm for 4 hours under treatment of aTc (final concentrations: 0, 10, 20, 50, 100 and 200 ng/μL). The fluorescence and absorbance (OD600) were measured using a plate reader. The fluorescence intensity of each RBS variant was divided by bacterial cell density. The data are presented as the means ± standard deviations based on three wells per group. Significant differences are shown as * P <0.05 (t-test). Abbreviations: aTC, anhydrotetracycline; OD600, optical density at 600 nm; RBS, ribosome-binding site; RFP, red fluorescent protein.

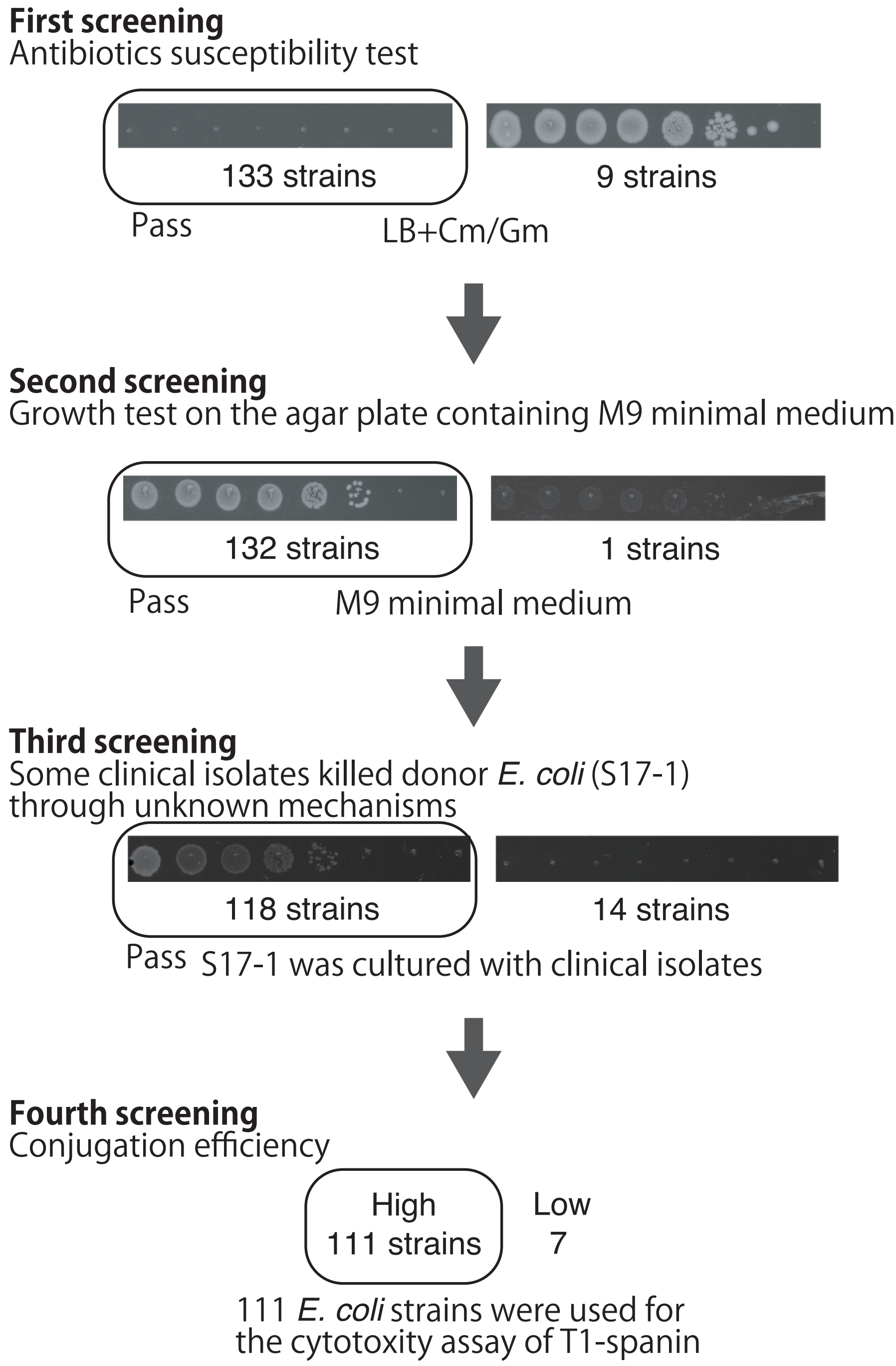

Fig. S3. The process of selecting clinical isolates for evaluating the bactericidal activity of T1-spanin is shown using the example of *Escherichia coli*.
In the first screening, an antibiotic susceptibility test was performed to select bacteria that were resistant to either chloramphenicol (Cm) or gentamicin (Gm), and clinical isolates that were resistant to both were excluded. In the second screening, the growth of the clinical isolates was evaluated in M9 minimal medium, which was used to inhibit the growth of S17-1. Clinical isolates that showed inhibited growth in M9 minimal medium were excluded. In the third screening, clinical isolates were co-cultured with S17-1 to evaluate the viability of each strain. Fourteen strains showed bactericidal activity against S17-1. Finally, a fourth screening was performed to select bacteria that underwent conjugation and had at least 100-fold more colonies than the control bacteria using a spot assay. Of the 142 clinical isolates of *E. coli*, 111 were used for the bactericidal evaluation assay of T1-spanin.

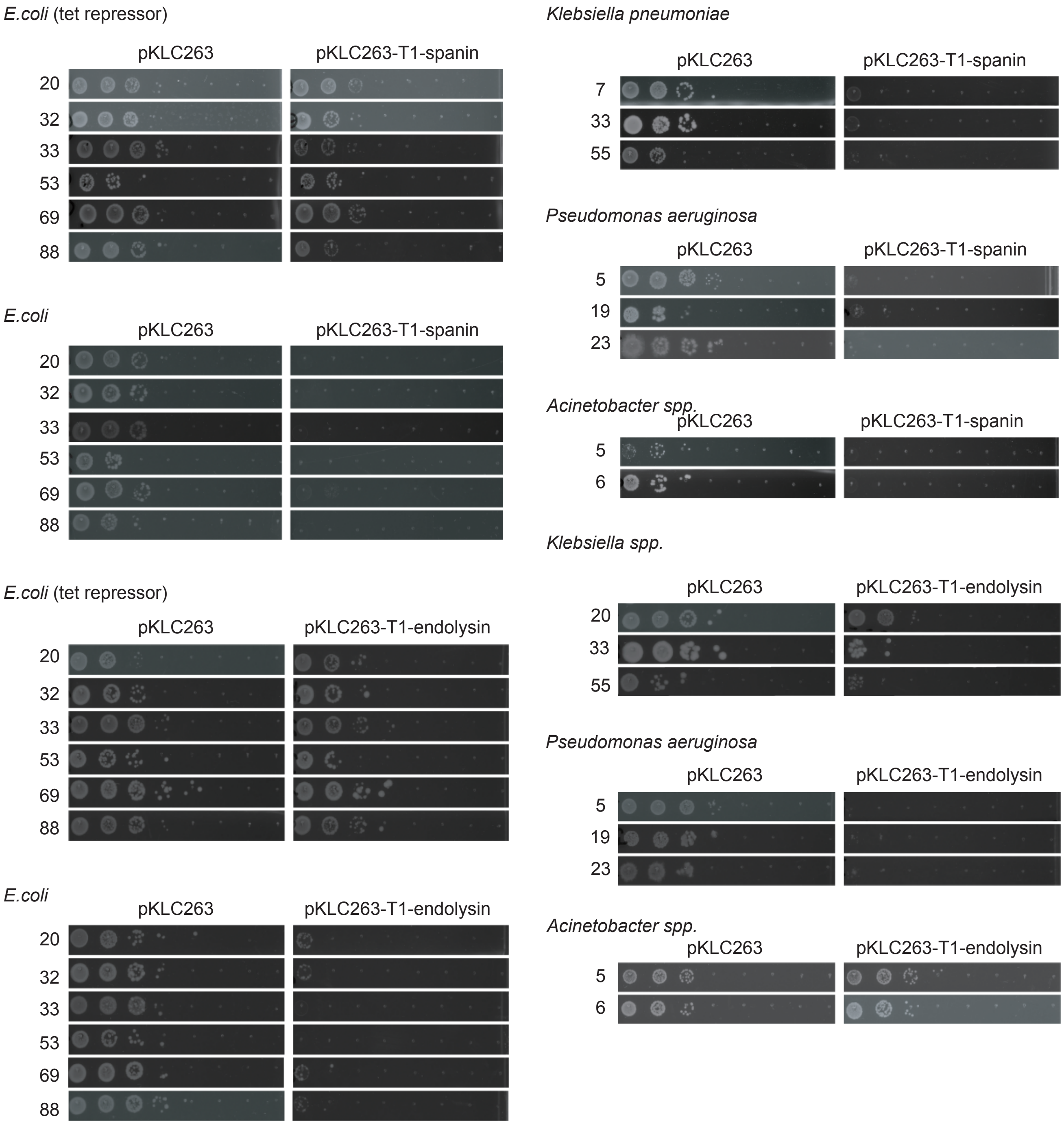

Fig. S4. Culture plates showing the results of T1-spanin and T1-endolysin killing studies. Clinical isolates, including *E. coli*, *Klebsiella* spp., *P. aeruginosa*, and *Acinetobacter* spp., were co-cultured with *E. coli* S17-1 carrying plasmids with T1-spanin or T1-endolysin (pKLC263 series) and serially diluted. The host bacteria were then spotted onto M9 minimal broth+Cm medium to examine the killing activity of T1-spanin and T1-endolysin. Abbreviation: Cm, chloramphenicol.
